# Direct evidence for activated CD8+ T cell transmigration across portal vein endothelial cells in liver graft rejection

**DOI:** 10.1007/s00535-016-1169-1

**Published:** 2016-02-18

**Authors:** Taro Kariya, Hisashi Ueta, Xue-Dong Xu, Daisuke Koga, Taichi Ezaki, Enqiao Yu, Satoshi Kusumi, Yusuke Kitazawa, Yasushi Sawanobori, Tatsuo Ushiki, Thomas Issekutz, Kenjiro Matsuno

**Affiliations:** 1 Department of Anatomy (Macro), Dokkyo Medical University, School of Medicine, Tochigi, Japan; 2 Department of General Surgery, Dalian Medical University, 1st Affiliated Hospital, Dalian, China; 3 Department of Microscopic Anatomy and Cell Biology, Asahikawa Medical College, Hokkaido, Japan; 4 Department of Anatomy and Developmental Biology, School of Medicine, Tokyo Women’s Medical University, Tokyo, Japan; 5 Department of Morphological Science, Kagoshima University Graduate School of Medical and Dental Sciences, Kagoshima, Japan; 6 Division of Microscopic Anatomy, Niigata University Graduate School of Medical and Dental Sciences, Niigata, Japan; 7 Department of Pediatrics, Dalhousie University/IWK Health Center, Nova Scotia, Canada

**Keywords:** Transmigration, T-cell, Portal vein endothelia, Vascular cell adhesion molecule-1, α4β1 integrin (VLA-4)

## Abstract

**Background:**

Lymphocyte recruitment into the portal tract is crucial not only for homeostatic immune surveillance but also for many liver diseases. However, the exact route of entry for lymphocytes into portal tract is still obscure. We investigated this question using a rat hepatic allograft rejection model.

**Methods:**

A migration route was analyzed by immunohistological methods including a recently developed scanning electron microscopy method. Transmigration-associated molecules such as selectins, integrins, and chemokines and their receptors expressed by hepatic vessels and recruited T-cells were analyzed by immunohistochemistry and flow cytometry.

**Results:**

The immunoelectron microscopic analysis clearly showed CD8β^+^ cells passing through the portal vein (PV) endothelia. Furthermore, the migrating pathway seemed to pass through the endothelial cell body. Local vascular cell adhesion molecule-1 (VCAM-1) expression was induced in PV endothelial cells from day 2 after liver transplantation. Although intercellular adhesion molecule-1 (ICAM-1) expression was also upregulated, it was restricted to sinusoidal endothelia. Recipient T-cells in the graft perfusate were CD25^+^CD44^+^ICAM-1^+^CXCR3^+^CCR5^–^ and upregulated α4β1 or αLβ2 integrins. Immunohistochemistry showed the expression of CXCL10 in donor MHCII^high^ cells in the portal tract as well as endothelial walls of PV.

**Conclusions:**

We show for the first time direct evidence of T-cell transmigration across PV endothelial cells during hepatic allograft rejection. Interactions between VCAM-1 on endothelia and α4β1 integrin on recipient effector T-cells putatively play critical roles in adhesion and transmigration through endothelia. A chemokine axis of CXCL10 and CXCR3 also may be involved.

**Electronic supplementary material:**

The online version of this article (doi:10.1007/s00535-016-1169-1) contains supplementary material, which is available to authorized users.

## Introduction

Immunosurveillance is conducted by recirculating lymphocytes and dendritic cells (DCs) [1]. These cells actively patrol each organ in the body by entering from the blood, and then transmigrating from the blood vessels into the tissue. Many studies have revealed that leukocyte trafficking involves sequential and synergistic cellular interactions between leukocytes and vascular endothelial cells via mediating specified molecules such as selectins, integrins, chemokines [2], and the recently identified diapedesis-associated molecules [3]. Thus, an appropriate expression of these relevant molecules in both cell types would be expected to regulate leukocyte positioning and the subsequent migratory pathway to enable effective immunosurveillance. However, if their expressions were to be dysregulated by pathological situations such as inflammation and infection, the result would be promotion of aberrant cell migration to the inflammatory site.

The liver is constantly subjected to antigen exposure in the portal venous blood derived from the gastrointestinal tract. To inspect these antigens, the liver is equipped with two distinct types of defense. One is the deployment of Kupffer cells and natural killer cells for rapid eradication of invaders, and the other is active patrolling by lymphocytes and DCs. Thus, the liver is extensively surveyed by these immunocompetent cells and their trafficking would be further enhanced, if inflammatory stimuli persisted in the liver in order to develop adaptive immune responses. In this respect, the portal tract have been considered as a central site for adaptive immune response of the liver diseases and identified as massive leukocyte accumulation sites in many types of liver disease such as hepatitis [4], infection [5], and transplant rejection [6]. Importantly, liver is sometimes lesioned unexpectedly by these infiltrates as bystander damages, which lead to hepatocellular necrosis, fibrosis, and liver failure [7]. Therefore it is important to know the exact route of migration and related mechanisms for this cellular trafficking to understand the pathogenesis of immune cell-mediated liver disease, however, it remains undetermined so far.

Previously we have investigated the transit time and migration pathway of recirculating T-cells and DCs in the normal liver [8, 9]. Both adoptively transferred T-cells and DCs showed similar trafficking patterns, appearing first in the sinusoid, then in the portal tract. These cells soon entered the draining lymphatics in the portal tract and migrated to the hepatic lymph nodes. Of note, we found that donor lymphocytes adhered to the wall of the portal vein (PV, both inside and outside of the basal lamina), suggesting the possibility of lymphocyte transmigration across the vessel walls of PV to reach the portal tract [8]. However, we could not investigate this phenomenon further because of the infrequent identification of this event in physiological settings.

In this study, by employing a rat liver allograft rejection model and a recently developed immunoelectron microscopic analysis method, we describe the first evidence that activated T-cells directly transmigrate across the endothelial cells of the PV and identify some of the molecules involved in this phenomenon.

## Materials and methods

### Animals

Inbred ACI (RT1A^a^B^a^) and Lewis (RT1A^1^B^1^) rats were supplied by Charles River Japan Inc. (Tsukuba, Japan). All animals were reared under specific pathogen-free conditions. Animal handling and care were approved by the Dokkyo Medical University Committee, in accordance with Dokkyo University’s Regulations for Animal Experiments and Japanese Government Law (No. 105). Male ACI rats served as liver graft donors, and the Lewis rats served as recipients, implanted at 8–10 weeks, with a body weight of 200–220 g. General anesthesia was used for surgery and euthanasia. Anesthesia was provided with isoflurane (Mylan Inc., Tokyo, Japan), administered with an isoflurane vaporizer (SN-487-OT, Shinano Manufacturing, Tokyo, Japan).

### Antibodies and reagents

All monoclonal antibodies (mAbs), polyclonal antibodies, and labeled secondary antibodies used are listed in Suppl. Table 1. Some mAbs were purified from culture supernatants and coupled to fluorescein isothiocyanate, R-phycoerythrin (Dojin, Kumamoto, Japan), Alexa flour^®^−488, −594, −647 (Molecular Probes, Eugene, OR), or PerCP Cy5.5 (Abcam plc, Cambridge, UK) in house.

### Orthotropic liver transplantation (LTx)

Orthotropic LTx was performed without anastomosis of the hepatic artery, as described [10]. Surgical procedures were performed under clean conditions in a laminar flow clean bench.

### Conventional scanning electron microscopy (SEM)

SEM was performed as described previously [8]. In brief, anesthetized rats were perfused through the ascending aorta with physiological saline followed by 2 % glutaraldehyde in a 0.1 M phosphate buffer (PB). After perfusion fixation, the liver was cut into small pieces, and further fixed with 2 % glutaraldehyde overnight at 4 °C. Tissue slices 100–200 μm thick were prepared by microslicer (DTK-2000, Dosaka EM CO., LTD. Kyoto, Japan), then incubated successively for 1 h with 1 % tannic acid, 0.1 M PB for rinsing, and 1 % OsO_4_ for conductive staining. The specimens were dehydrated in a graded ethanol series, transferred to isoamyl acetate and dried in a critical point dryer (HCP-2, Hitachi Koki Co. Ltd, Tokyo, Japan) using liquid NO_2_. Dried samples were mounted onto aluminum stubs, coated with platinum–palladium in an ion-sputter coater (E1010, Hitachi, Tokyo, Japan), and observed in a field-emission SEM (S4300N Hitachi).

### Immuno-SEM

Rats were first perfused with saline as above followed by 4 % paraformaldehyde in a 0.1 M PB. The graft liver was then excised and cut into small pieces of about 1 cm on all sides. Liver sections 100 µm thick were cut with the microslicer, rinsed with phosphate-buffered saline (PBS) for 10 min, and incubated with 2 % normal goat serum for 30 min. The sections were incubated with anti-rat CD8β mAb for 12 h, rinsed three times with PBS, and subsequently incubated with 10 nm colloidal gold conjugated goat polyclonal anti-mouse IgG (BBI Solutions, Cardiff, UK) for 1 h. After being rinsed, sections were incubated in the gold-enhancing solution (Goldenhance™ EM; Nanoprobes, NY, USA) for 10 min, to enlarge the labeled gold particles for SEM [11]. Subsequently, sections were rinsed with PB for 10 min, conductive stained as described above, dehydrated with a graded ethanol series, and dried in a critical point dryer. Dried specimens were mounted as above, coated with platinum–palladium at a thickness of about 5 nm in the ion-sputter coater, and observed in a field-emission SEM (SU 70 Hitachi). The signals were also detected with backscatter electron (BSE) imaging.

### Transmission electron microscopy (TEM)

Graft liver was perfused with 2 % paraformaldehyde in PBS. Excised liver was further fixed with sequential incubation with 1 and 2 % glutaraldehyde in PBS at 4 °C, overnight. Sections were prepared and post-fixed with 1 % osmium tetroxide, electron-stained with 3 % uranyl acetate, and embedded in an Epon-Araldite mixture. Ultrathin sections (approximately 60 nm) were examined under a Hitachi H-7000 electron microscope (Hitachi, Tokyo, Japan). Serial semi-thin sections (approximately 500 nm) were stained with toluidine blue for light microscopy.

### Immunohistochemistry

To evaluate expression profiles for several adhesion molecules and chemokines on liver endothelia, graft livers were successively isolated after LTx and embedded in OCT. To label proliferating cells, recipient rats received an intravenous injection of BrdU (6 mg/200 g body weight) in sterile PBS 1 h before killing. Multiple immunoenzyme or immunofluorescence staining of cryosections 4 μm thick was performed as described previously [10]. For immunofluorescence staining, images were captured with an Axioskop2 Plus microscope with an AxioCam MRm camera (Carl Zeiss, Jena, Germany). To depict the tissue framework more clearly, the original blue image from type IV collagen staining was converted to white pseudocolor with Axiovision software (Carl Zeiss).

### Flow cytometric (FCM) analysis

Recipient blood cells within the graft circulation were isolated by gentle perfusion with 50 ml of HBSS containing 2 mM EDTA, 10 U/ml heparin, 100 μg/ml DNase I, and 5 % fetal calf serum from the PV at 3 ml/min. Single-cell suspensions were prepared by Lympholyte^®^ density gradient (Cedarlane, Canada). After incubation with a donor class I major histocompatibility complex (MHCI) mAb and secondary anti-mouse IgG microbeads, recipient cells were further magnetically purified by depleting donor cells using autoMACS (Miltenyi Biotec K. K., Tokyo, Japan). After FcγIIR blocking, the cells were subjected to four-color immunofluorescent staining. Stained cells were acquired on a FACSCalibur (BD Biosciences, Franklin Lakes, NJ) and data were analyzed with FlowJo ver. 9.2 (FlowJo LLC, Ashland, OR).

### Statistical analysis

Statistical analysis was performed using Student’s *t* test.

## Result

### Immune cell kinetics after LTx

**Fig. 1:**
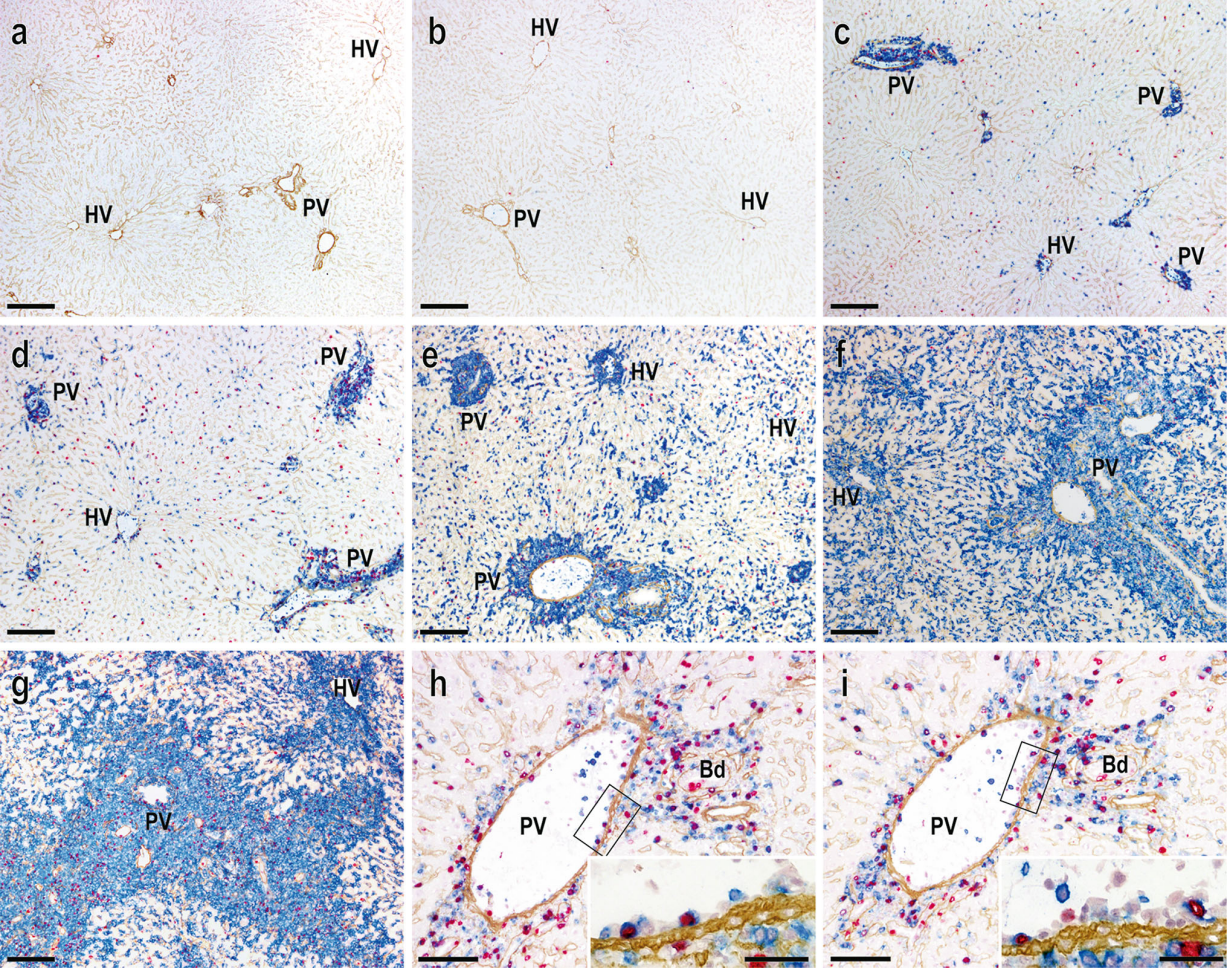
Kinetics of recipient cell infiltration into the graft after transplantation. Triple immunoenzyme staining by recipient MHCI (*blue*), type IV collagen (*brown*) and BrdU (*red*) in control liver (**a**), at 1d (**b**), 2d (**c**), 3d (**d**), 5d (**e**), 7d (**f**) and 10d (**g**) after LTx. Note recipient MHCI^+^ cell infiltration around the PV (PV) was dominant compared to the hepatic vein (HV) area over time. **h**, **i** Recruitment of TCRαβ^+^ (**h**, *blue*) or CD8β^+^ (**i**, *blue*) cells to portal tract on day 4, depicted by serial sections. Many TCRαβ^+^ or CD8β^+^ cells were seen in the portal tract and some of them were actively proliferating by incorporating BrdU. Some cells attached to the PV vessel walls (*inset* of **h** and **i**). Representative figures of three rats. *Bd* bile duct, *PV* portal vein, *HV* hepatic vein. *Scale bars*: **a**–**g** 200 μm; **h**, **i** 100 μm; *inset* of **h** and **i** 50 μm

In this transplantation setting, donor graft was acutely rejected by ~11 days [10]. In the graft, recipient MHCI-positive cells were almost absent on day 1 and appeared from day 2 and progressively increased. Notably prominent recipient cell infiltration was observed in the portal tract, which gradually expanded by the infiltrated cells (Fig. 1a–g). At the late stage, the sinusoidal area decreased to ~30 % of the total surface area (Fig. 1g) [6]. The majority of infiltrating cells on day 4 were positive for T-cell receptor (TCR)αβ, CD8β (Fig. 1h, i), or CD4 (not shown) with a high labeling index of BrdU, suggesting that infiltrated cells were mostly activated T-cells. These cellular kinetics results were consistent with a previously reported LTx model using a similar MHC-disparate combination, in which effector T-cell recruitment led to acute rejection [6].

### Transmigration of CD8β^+^ T-cells across the vessel walls of PV

**Fig. 2:**
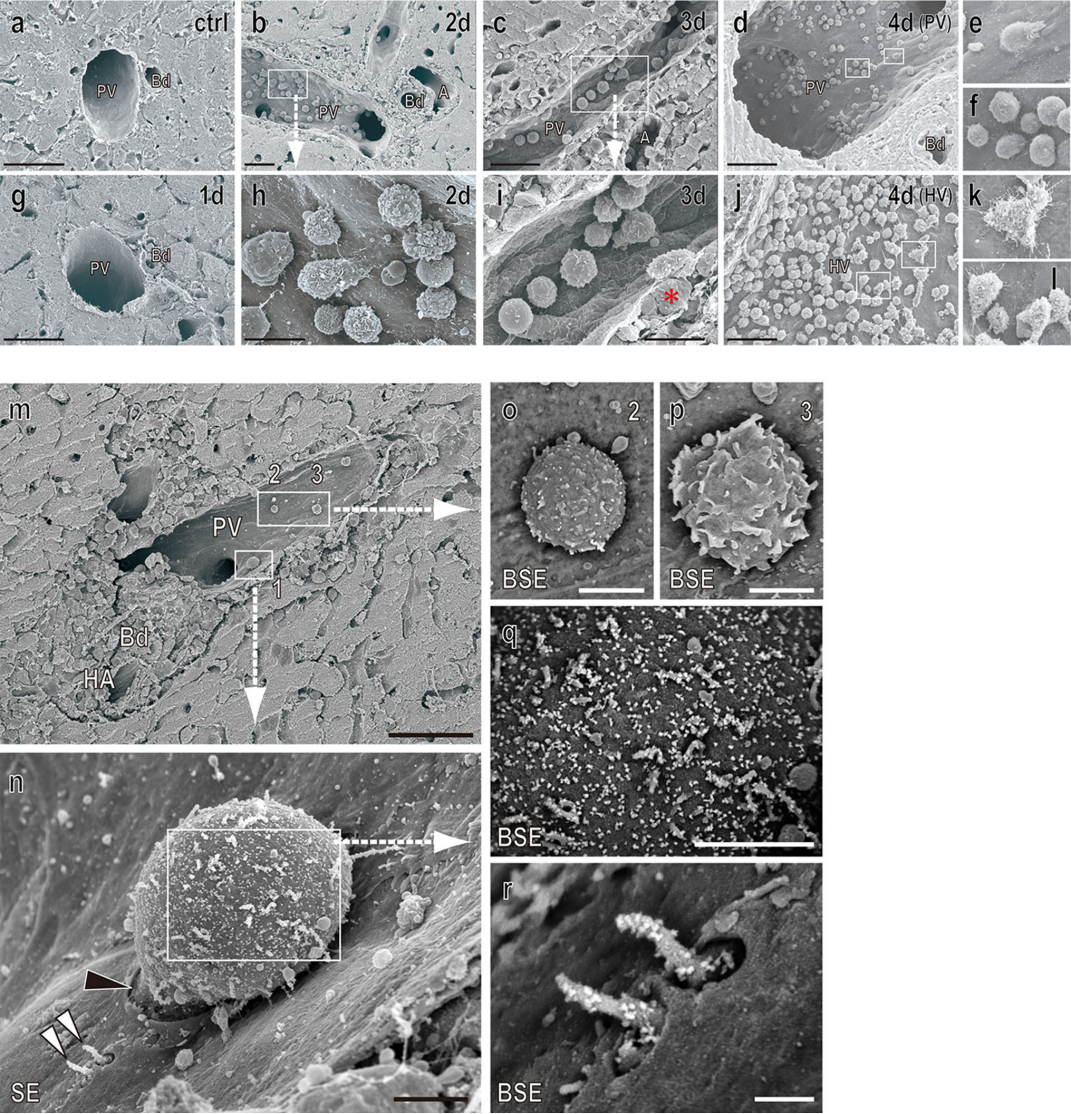
SEM images of the portal tract of the allograft. Representative SEM images of the PV (**a**–**i**) and hepatic vein (**j**–**l**) after LTx. Note the appearance of adherent cells from day 2 (**b**, **h**) in Fig. 1. Note poorly polarized cells, with a less protrusional shape of adherent cells at the PV (**e**, **f**) compared to those of hepatic vein (**k**, **l**). Immuno-SEM analysis for CD8β (**m**–**r**). Note CD8β^+^ cells undergoing transmigration at the PV (**m** and **n**, *black arrowhead*). A backscatter electron (BSE) image of 1–3 (**o**–**r**). Note considerable amount of CD8β signal was evenly and densely distributed in the cell body (**q**) as well as the lamellipodia-like structure (**r**, *white arrowheads*) of 1, but not 2 and 3 (**o** and **p**). *Bd* bile duct, *PV* portal vein, *HA* hepatic artery, *HV* hepatic vein. *Scale bars*: **a**, **c**, **d**, **j**, and **m** 50 μm; **b**, **g** 30 μm; **h**, **i** 10 μm; **n**–**p** 2.5 μm; **q** 2 μm; **r** 0.5 μm

Immunohistochemical analysis showed that some cells attached on the wall of the PV (Fig. 1h, i). SEM imaging of the allograft showed that the number of leukocytes contacting the vessel wall gradually increased from day 2 at the portal tract (Fig. 2a–i). Of interest, their shapes were obviously different from those in the hepatic vein, with a spherical, non-polarized morphology (Fig. 2d–f) compared to a non-spherical morphology with spreading microvilli in the latter (Fig. 2j–l). Many bulges were also formed on the vessel wall compared to the control group, implying the presence of migrating lymphocytes underneath the endothelial sheet (red asterisk, in Fig. 2i). Furthermore, by immuno-SEM analysis using the anti-CD8β mAb followed by nano-gold–conjugated secondary antibody, we could detect CD8β^+^ particles on a cell that was just passing through the PV endothelial cell (Fig. 2m, n, q, and r). We could not investigate their transmigration of CD4^+^ T-cells because of a lack of anti-rat CD4 mAb compatible with 4 % paraformaldehyde fixation, an essential procedure for immuno-SEM analysis.

### T-cell transmigration through endothelial cells of the PV

**Fig. 3:**
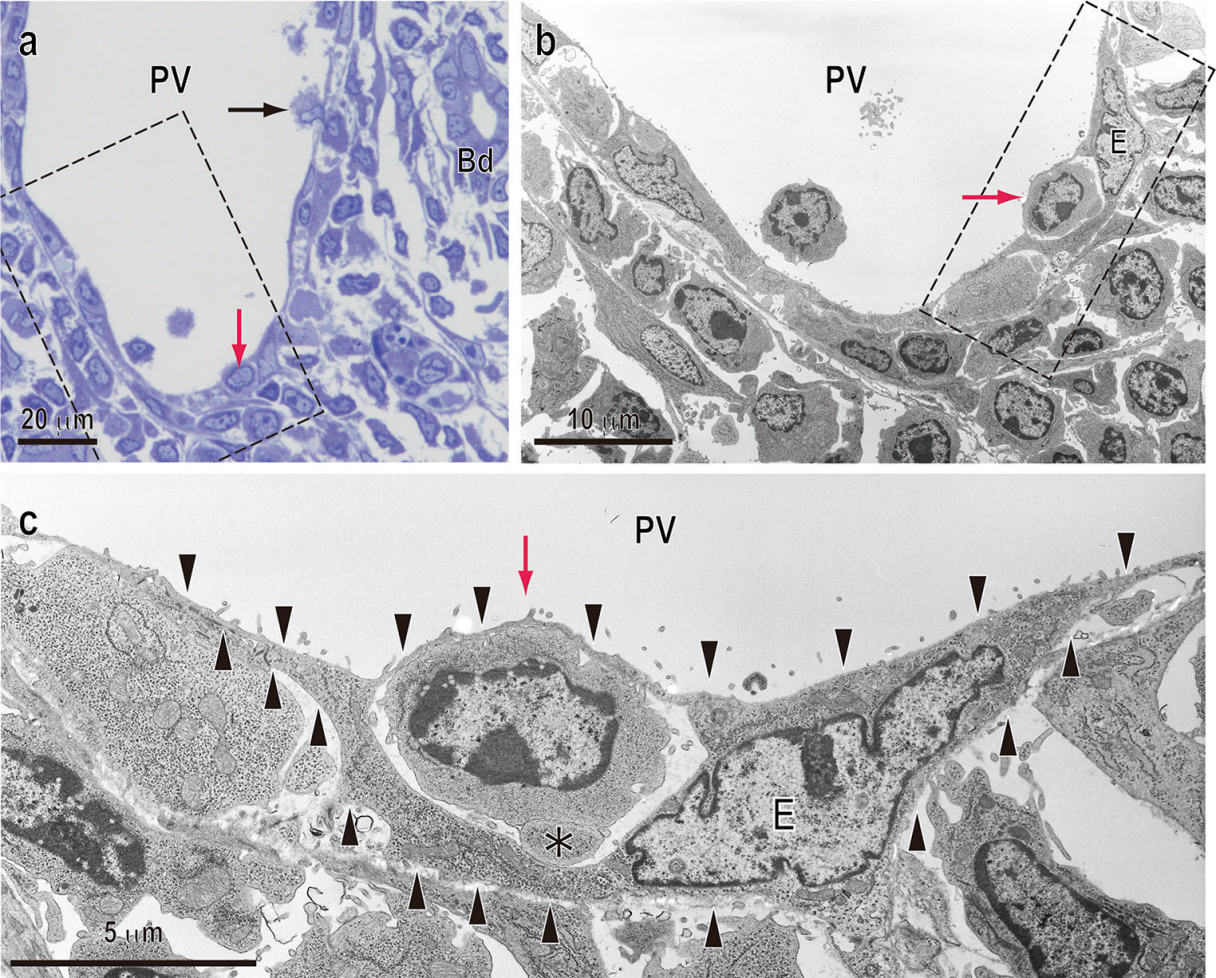
TEM image of T-cell transmigration through endothelial cells of the portal vein. **a** Toluidine blue staining of a semi-thin-section of the portal tract on day 3 after LTx. *Red or black arrows* indicate transmigrating mononuclear cells. **b** TEM image of serial sections (*dotted line* area in **a**). **c** Magnified TEM image in *dotted line* area in **b**. Note a mononuclear cell, probably a lymphocyte (*red arrow*), inside an endothelial cell (*E*: outlined by *arrowheads*). Also note some protrusion at the leading edge (*asterisks* in **c**). *Bd* bile duct, *PV* portal vein. *Scale bars*: **a** 20 μm; **b** 10 μm; **c** 5 μm

Light microscopy of semi-thin-sections of the day 3 graft showed binding of several blood cells to the walls of large vessels. Anatomically, these vessels were defined as PVs by the presence of bile ducts in their vicinity (Fig. 3a). TEM observation of serial sections clearly revealed a mononuclear round cell, probably a lymphocyte, passing through an endothelial cell of the PV (Fig. 3b, c). It is noteworthy that its migrating pathway seemed to be a transcellular route, in which migrating cells directly penetrate through the endothelial cell body, rather than an intercellular route. In addition, lamellipodia-like structures were also detected at the leading edge (Fig. 3c, asterisk).

### Expression of cell migration-associated molecules on graft endothelial cells

**Fig. 4:**
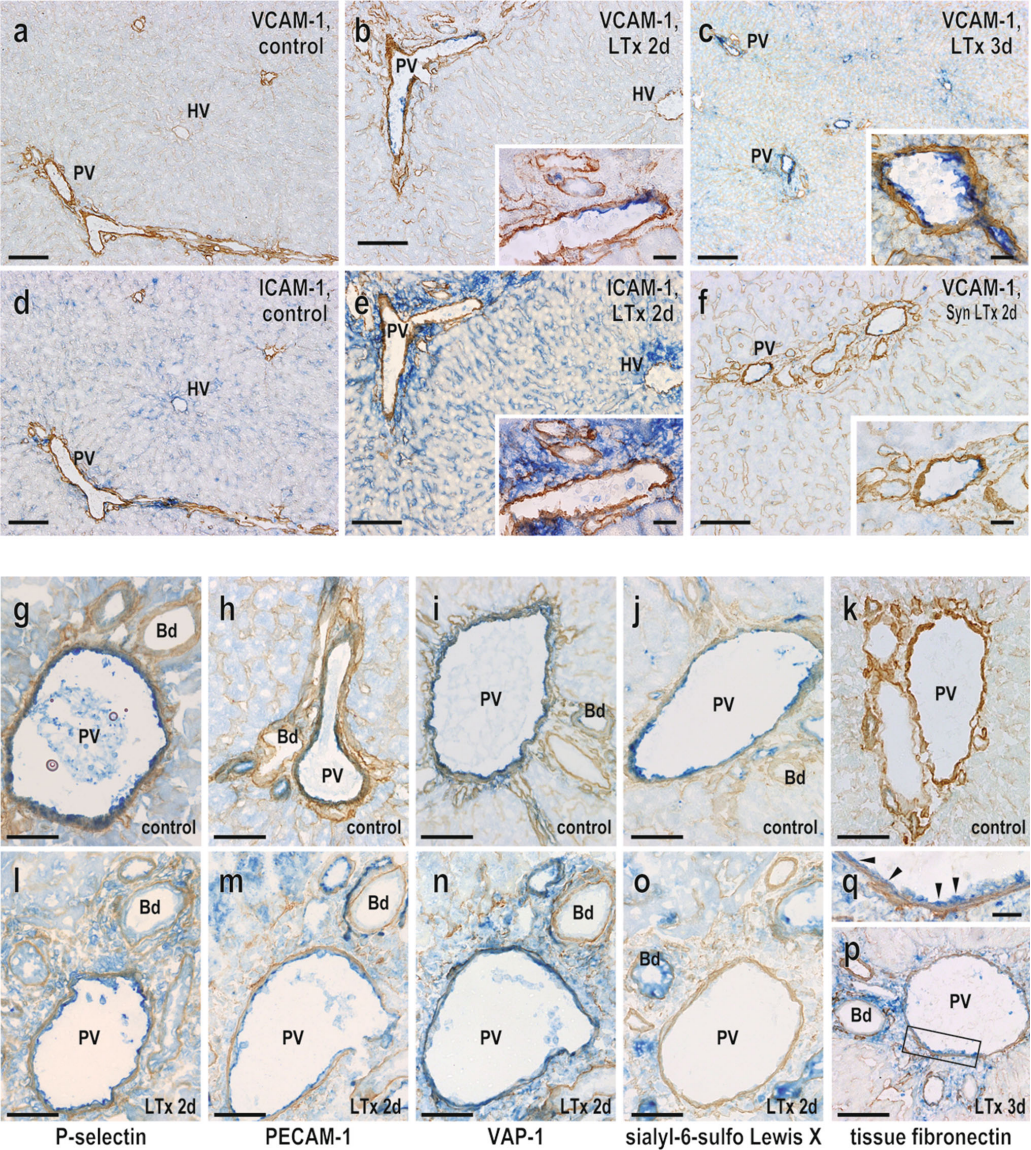
Immunohistochemical analysis of cell migration-associated molecule expression in liver allograft endothelia. VCAM-1 and ICAM-1 expression was upregulated after LTx (**a**–**e**). Note reciprocal expression pattern for VCAM-1 and ICAM-1 at portal vein and sinusoidal endothelia, respectively (b versus e). VCAM-1 expression was slightly induced in the syngeneic graft (**f**). The expressions of P-selectin (**g** and **l**), PECAM-1 (**h**, **m**), VAP-1 (**i**, **n**) and tissue fibronectin (**k**, **p**, and **q**, *arrowhead*) was upregulated while sialyl-6-sulfo-Lewis X (**j**, **o**) declined at the PV endothelia after LTx. *Scale bars*: **a**, **c**, and **d** 200 μm; **b**, **e**, and **f** 100 μm; *inset* of **b**, **c**, **e**, and **f** 20 μm; **g**–**p** 100 μm; **q** 20 μm

Next, we analyzed the expression of cell migration-associated molecules on the graft vascular endothelium by immunohistochemistry. Several cell migration-associated molecules were expressed constitutively and selectively on the PV endothelia in normal donor livers as shown in Table 1. From day 2 after LTx, vascular cell adhesion molecule-1 (VCAM-1) was preferentially induced on the portal but not sinusoidal endothelia and persisted thereafter (Fig. 4a–c). Occasionally, endothelia of the central vein were also partly positive for this molecule. Intercellular cell adhesion molecule-1 (ICAM-1) expression was also upregulated after LTx but was restricted to sinusoidal and hepatic vein endothelia (Fig. 4d, e). Weak expression of VCAM-1 in the PV (Fig. 4f) and of ICAM-1 on sinusoidal endothelia (not shown) was occasionally seen in the syngeneic graft on day 2. *P*-selectin, platelet/endothelial cell adhesion molecule (PECAM)-1, and vascular adhesion protein (VAP)-1 were constitutively expressed and slightly upregulated in the PV endothelia after LTx (Fig. 4g–i, l–n). In contrast, the expression of sialyl-6-sulfo Lewis X (CD15s), a ligand of selectins, was downregulated in the PV endothelia from day 2 after LTx (Fig. 4j–o). Tissue fibronectin (tFN), one of the ligands of α4β1 integrin, which is upregulated after LTx [12], was temporarily expressed in the PV endothelia from day 3 after LTx (Fig. 4k, p and q). E-selectin expression was unchanged, and neither mucosal vascular addressing cell adhesion molecule-1 (MAdCAM-1) nor ICAM-2 was detected on the surface of the graft vasculature (not shown). The spatio-temporal expression profile of these molecules is summarized in Table 1.

**Table 1:** Expression of cell migration-associated molecules on liver endothelium after transplantation

Molecules	Area	Days after LTx						
		Control	1	2	3	4	5	2 (Syn)
E-selectin (CD62E)	PV	+	+	+	+	+	+	NT
	SN	−	−	−	−	−	−	NT
	HV	−	−	−	−	−	−	NT
P-selectin (CD62P)	PV	+	++	++	+	+	+	NT
	SN	−	±	−	−	−	−	NT
	HV	+	+	+	+	+	±	NT
Sialyl-6-sulfo-Lewis X (CD15s)	PV	+	+	±	−	−	−	NT
	SN	−	+	+	+	±	−	NT
	HV	+	+	+	+	+	+	NT
PECAM-1 (CD31)	PV	+	++	++	++	++	++	NT
	SN	−	−	−	−	−	−	NT
	HV	−	±	±	±	±	±	NT
ICAM-1 (CD54)	PV	−	−	−	−	−	−	−
	SN	±	+	+	++	±	±	+
	HV	−	−	±	±	±	±	±
VCAM-1 (CD106)	PV	−	−	+	++	++	++	±
	SN	−	−	−	−	−	−	−
	HV	−	−	±	±	±	±	±
MAdCAM-1	PV	−	−	−	−	−	−	NT
	SN	−	−	−	−	−	−	NT
	HV	−	−	−	−	−	−	NT
VAP-1	PV	+	+	++	++	+	+	NT
	SN	−	−	−	−	−	−	NT
	HV	±	+	+	+	+	+	NT
Tissue fibronectin (tFN)	PV	−	−	±	+	NT	NT	NT
	SN	−	−	−	−	NT	NT	NT
	HV	−	−	−	−	NT	NT	NT

Double immunohistochemical analysis was performed in Fig. 4 and the intensity of expression was scored from days 1–5 in three distinct liver graft compartments: the portal vein (PV), sinusoid (SN), and hepatic vein (HV) endothelia, each summarized. Staining intensity was evaluated as − (negative), ± (faintly/slightly positive), + (positive), and ++ (strongly positive): *n* = 3 rats. *NT* not tested, *Syn* syngeneic LEW to LEW LTx

### Expression of cell migration-associated molecules on recruited T-cells in the graft vasculature

**Fig. 5:**
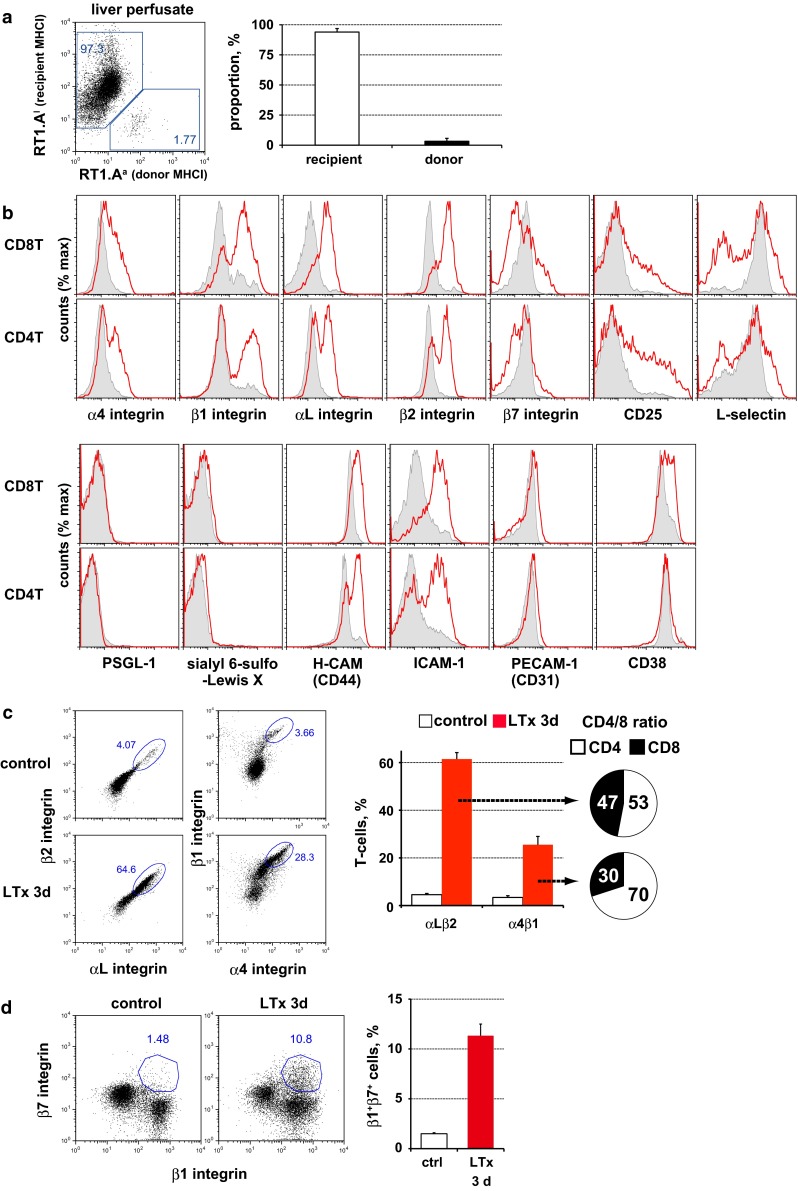
Flow cytometry analysis of recruited T-cells in the graft vasculature. **a** Purity of liver perfusate analyzed. **b** Expression of migration-associated molecules in CD4 and CD8 T-cells in the liver perfusate at day 3 after LTx (*red line*) and in control (*filled gray*). **c** Upregulation of αLβ2 integrin and α4β1 integrin on activated T-cells after LTx. **d** Induction of β7 integrin in β1 integrin^high^ T-cells. Representative figures of more than three experiments

To confirm the expression of cell migration-associated molecules in recipient migrating cells, recipient cells inside the graft vasculature were isolated and analyzed by multicolor FCM (Fig. 5). Recipient MHCI^+^ cells were about ~95 % of the population (Fig. 5a). Histological analysis of the perfused liver indicated that the tissue structure was preserved and the infiltrated cells were not washed away from the interstitial area when compared with unperfused tissue. This result suggested that cells in perfusate were obtained mostly from the blood within the graft and that contamination from cells that had already extravasated was minimal (Suppl. Fig. 1a). Recipient cells in the perfusate consisted of T-cells (~30 %), neutrophils (~30 %), macrophages (~20 %), B-cells (~5 %), and natural killer cells (~5 %) (Suppl. Fig. 1b). Recipient T-cells showed a typical activated-cell phenotype with CD25, CD44H and ICAM-1 upregulated and CD62L downregulated (Fig. 5b). In addition, expression of α and β integrins was increased in both CD4 and CD8 T-cells. Of note, almost 60 % of T-cells were αLβ2^+^ and 25 % were α4β1^+^ (Fig. 5c). Interestingly, α4β1 expression was dominant in the CD4 subset (CD8 30 %, CD4 70 %, Fig. 5c). In addition, some β1 integrin^+^ cells significantly co-expressed β7 integrin (Fig. 5d). On the other hand, P-selectin glycoprotein ligand (PSGL)-1, CD15s (Fig. 5b), and α5 integrin (not shown) were not induced in T-cells after LTx. Diapedesis-associated molecules CD38, but not PECAM-1 (CD31) was slightly up-regulated in CD8 T-cells. CD4 T-cells did not express both molecules.

### Expression of chemokines and chemokine receptors in the grafts

**Fig. 6:**
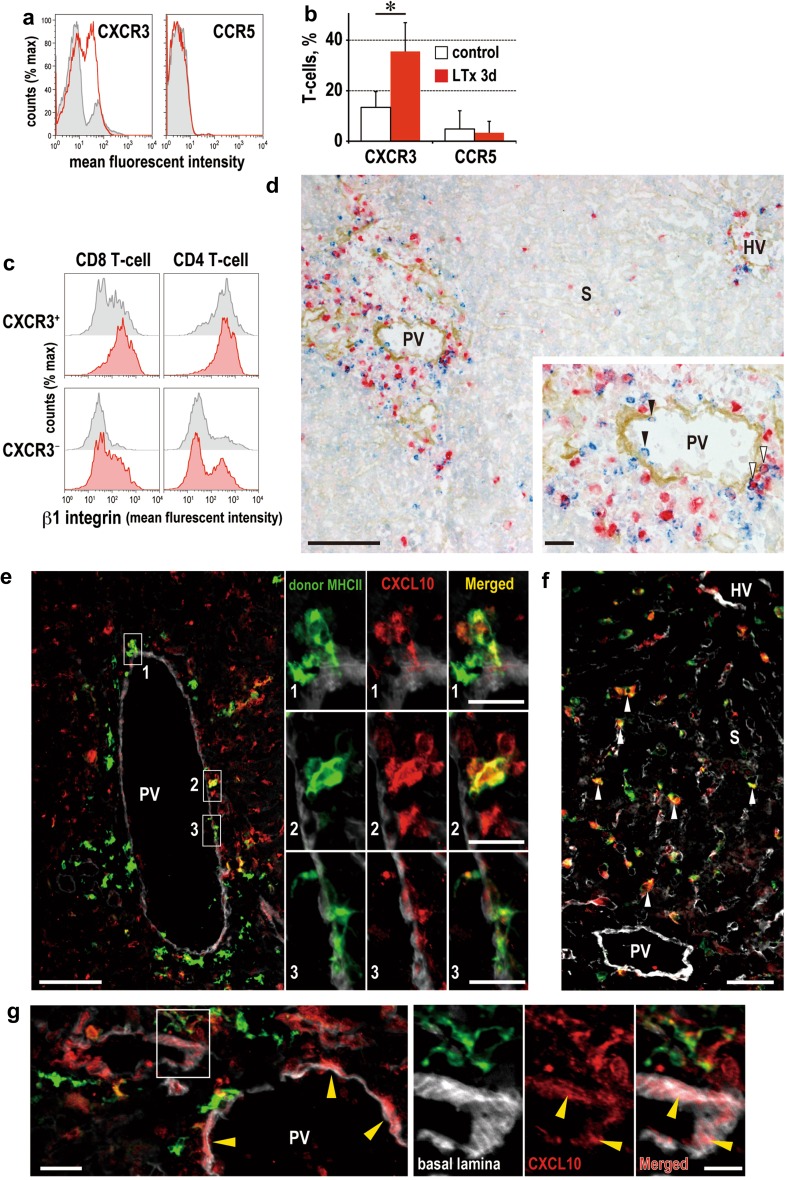
Expression of chemokines and chemokine receptors expression after LTx. CXCR3 expression in liver perfusate at day 3 after LTx (**a**, **b**).  *p < 0.05. Note that high β1 integrin expression was preferentially induced in CXCR3^+^CD8 T cells after LTx (**c**, *filled red*) compared with control (**c**, *filled gray*). Preferential migration of CXCR3^+^ cells (*blue*) in portal tract in a day 3 allograft (**d**). Note attaching CXCR3^+^ cell in the PV endothelial wall (*black arrowhead in inset*). Some CXCR3^+^ cells were actively proliferating (*white arrowhead in inset*). Triple immunofluorescence staining with CXCL10 (*red*) and type IV collagen-like structure (*white pseudocolor*) with donor MHCII (**e** and **g**, *green*) or CD163 (**f**, *green*) in a day 3 allograft. Note co-localization of CXCL10 in donor MHCII^high^ cells in the portal tract (**e**) as well as CD163 in the sinusoid (**f**, *white arrowhead*). Also note the deposition of CXCL10 in the PV vessel wall (**g**, *yellow* arrowheads). *Scale bars*: **d** and **e**, 100 μm; *inset* of **d** and **e**, 20 μm; **f**, 50 μm; **g**, 20 μm; *inset* of **g**, 10 μm

First, we investigated expression of chemokine receptors in activated T-cells in the perfusate. Th1-related chemokine receptor CXCR3 but not CCR5 (Fig. 6a, b) or CXCR6 (not shown) was significantly upregulated in the LTx group compared to controls. Notably more β1 integrin^high^ T-cells were seen in the CXCR3^+^ population than in their CXCR3^−^ counterparts in LTx group. In particular, CXCR3^+^CD8β^+^ T-cells upregulated β1 integrin, in which a proportion of β1 integrin^high^ cells was 79.7 ± 5.8 % in the LTx liver perfusates compared to 42.0 ± 6.3 % in the controls. CXCR3^+^CD4^+^ T-cells, defined by CXCR3^+^TCRαβ^+^CD8β^−^ population, constitutively expressed high levels of β1 integrin (Fig. 6c). Immunohistochemistry of day 3 graft livers also showed the expression of CXCR3 by migrated cells in the portal tract and those in the immediate vicinity of the PV endothelia (Fig. 6d). CCR9, known as a gut-homing molecule [13], was not detected (not shown).

Expression of CXCR3 ligands was further analyzed by immunofluorescent microscopy. CXCL10 expression was diffusely upregulated after LTx. Of interest, it was expressed not only in sinusoidal CD163^+^ Kupffer cells (Fig. 6f) but also in donor MHCII^high^ cells just underneath the PV endothelia (Fig. 6e). Furthermore, CXCL10 expression was sporadically detected in the basement lamina of the PV, indicating deposition of this chemokine, which was secreted by cells in the portal tract (Fig. 6g). CXCL9 was expressed constitutively in the hepatic vein area and did not change after LTx. Some CXCL9 expression was seen in α_M_ integrin^+^ neutrophils in the sinusoid after LTx (not shown).

## Discussion

By employing a recently improved immuno-SEM method [11], we have produced the first direct evidence of CD8^+^ T-cell transmigration through the PV in vivo. Interestingly, the TEM analysis revealed a unique transmigration pathway. Immunohistochemistry and FCM revealed that local expressions of VCAM-1 and tFN in the PV endothelia and their cognate ligand, α4β1 integrin, in activated T-cells could be responsible for this phenomenon. The chemokine CXCL10 and CXCR3 axis might also be involved.

Recipient T-cell infiltration was first observed on day 2 after LTx, preceded by generation of alloreactive T-cells that began in the secondary lymphoid organs 36 h after transplantation [10]. FCM analysis revealed that recipient T-cells in the perfusates of day 3 grafts upregulated both ICAM-1 and CD44 H, indicating the engagement of TCR signal in these cells. Thus, the recruited T-cells in the graft were mainly activated T-cells.

By FCM, we found the upregulation of αLβ2 (LFA-1) and α4β1 (VLA-4) integrins in activated T-cells in the LTx perfusate. While by immunohistochemistry, cognate ligands for αLβ2 and α4β1 integrins were differentially expressed in the graft endothelium, ICAM-1 in sinusoids, and VCAM-1 in PVs, respectively. Thus, T-cell migration behavior is likely to be partly regulated by integrin expression profiles, as reported in mouse Con-A hepatitis where distinct T-helper cells were selectively recruited [14]. In this study, we observed few transmigrating cells crossing the PV before day 2 after LTx, when a considerable number of recipient T-cells accumulated in the portal tract. This time lag might indicate that αLβ2 integrin^+^ T-cells first extravasate at the sinusoidal area and then migrate to portal tract via Disse’s space [8, 9]. These preceding infiltrates might induce chemokine secretion by donor resident cells or themselves and activate the PV endothelia, leading to modification of their adhesion molecules. These events should promote the subsequent direct transmigration through the PV. Although the upregulation of P-selectin expression was also detected in the PV endothelia, its cognate ligands such as PSGL-1 and CD15s were not induced in migrating T-cells. Conversely, although CD44H, a ligand for E-selectin [15], was upregulated in T-cells, E-selectin in the PV endothelial cells was unchanged. A previous in vitro study showed that the integrin α4β1–VCAM-1 interaction does not require a selectin-mediated signal on tethering or rolling under shear flow [16]. Accordingly, selectin involvement may be unnecessary in activated T-cell recruitment to the PV endothelium, as in the case of sinusoid [17]. Because VCAM-1 was also induced, albeit weakly, in the day 2 syngeneic graft as well as in a 24-h rat cardiac allograft [18], its expression was at least a partly antigen-independent process, i.e., an ischemia–reperfusion injury, an unavoidable complication following transplantation.

Interestingly, some T-cells co-expressed both β7 and β1 integrins. Because allosensitization of recipient T-cells induced by migrated donor DCs occurs systemically in the secondary lymphoid organs [10], alloreactive T-cells must be generated also in the gut-associated lymphoid tissue. In this instance, activated T-cells are known to predominantly upregulate α4β7 integrin [13]. Although MAdCAM-1, to which β7 integrin^+^ T-cells bind was not detected even after LTx, the β7 integrin molecule might act as an alternative VCAM-1 ligand [19] by which β7 integrin^+^ T-cells could bind to the PV.

tFN, another α4β1 integrin interacting molecule and also a ligand of CD44H, is induced in several transplant settings. The tFN–α4β1 integrin interaction induces several matrix proteases such as MMP-9, which promote leukocyte recruitment at the inflammatory site. Although tFN expression was sporadic and delayed by 1 day compared to that of VCAM-1, tFN–α4β1 integrin and/or tFN–CD44H interactions may have some role in activated T-cell positioning at the PV vessel walls.

VAP-1 was also selectively expressed by the PV endothelium. Blockade of VAP-1 by a specific antibody or by inactivation of its enzymatic function significantly decreases cellular infiltration into the portal tract in rat LTx [20] and in vitro transmigration activity of human peripheral blood leukocytes, respectively [21]. It is likely that VAP-1 would have a critical role in transmigration for some T-cell subsets that express Siglec-10 on activation [22] to across the PV.

Taken together, these results suggest that VCAM-1 and tFN in the PV endothelia and α4β1 integrins in T-cells are crucial for direct transmigration through the PV after LTx. VAP-1 may also play a role.

Our data showed that recruited T-cells significantly expressed the Th1-related chemokine receptor CXCR3 but not CCR5. β1 integrin^high^ expression correlated with CXCR3 expression in both CD4^+^ and CD8^+^ T-cells, suggesting that the upregulation of β1 integrin is linked to CXCR3-mediated signaling, known as the inside-out signal. On the other hand, CXCL10 was expressed by donor MHCII^high^ cells residing just beneath the PV endothelia and was obviously deposited in the PV endothelia, which may suggest local production and deposition of CXCL10 in the portal tract. We recently reported that a donor hepatic DC subset with the MHCII^high^CD103^+^CD11b^+^CD172a^+^ phenotype only partly migrates to recipient secondary lymphoid organs as passenger cells and some remains mainly in the portal tract after LTx. Soon after LTx, these DCs induce a strong proliferative response to migrated recipient T-cells in the portal tract [6]. An in vitro study by Curbishley et al. suggested that CXCR3 plays role in effector T-cells not only for binding to endothelium but also for diapedesis [17]. Taken together, we conclude that donor DCs remaining at the portal tract after LTx might produce and secrete CXCL10, which may deposit on the PV endothelia, thus promoting direct transmigration across the PV.

CCR5 is a marker of memory T-cells and is expressed by effector T-cells infiltrating around portal tract in murine graft-versus-host disease model at the chronic stage [23]. CCR5^+^ T-cells are probably a minor population in acute graft rejection as in the present study.

In physiological immunosurveillance, most leukocytes transmigrate across the intercellular gaps of endothelia, which is called paracellular migration. In contrast, the present TEM analysis showed that some cells transmigrated through the endothelial cell body. This type of transmigration may be called “transcellular migration” [3], which is known to occur in highly inflamed situations [24].

Recent studies have suggested active roles for endothelial cells in leukocyte transmigration [3]. Signals generated by molecular interaction with leukocytes elicit dynamic changes in endothelial cells, such as openings of the intercellular junction and trafficking of the lateral border recycling compartment, which is a unique membranous structure surrounding leukocytes during diapedesis [3]. In this respect, endothelial ICAM-1 is generally thought to play critical roles not only in leukocyte polarization and crawling in the diapedesis step but also in active endothelial change in the transmigration step, regardless of migration type [3]. Interestingly, Abadier et al. recently reported transcellular migration of CD4^+^ effector/memory T-cells through the inflamed endothelia of the blood–brain barrier in the absence of endothelial ICAM-1 and ICAM-2 [25]. In addition, they showed that the morphology of effector T-cells attaching to inflamed ICAM-1 or -2-deficient endothelia was poorly polarized, with a less protrusional shape. These characteristics were quite similar to our present results and suggest a common occurrence of this unique type of ICAM-independent transmigration in certain inflammatory situations.

Further study with advanced intravital imaging technique such as multi-photon laser scanning microscopy [26] would enable us to directly visualize the transmigration, which provide additional insights to clarify and understand this phenomenon.

In conclusion, we provide the first direct evidence of T-cell transmigration across PV endothelial cells during hepatic allograft rejection. Interactions between VCAM-1 and CXCL10 expressed in endothelia and α4β1 integrin and CXCR3 in recipient T-cells potentially play critical roles in this process. We conclude that the PV transmigration pathway could serve as a bypass to reach the affected area of the liver more rapidly during liver inflammation. α4β1 integrin^+^ T-cells, at least some CD8^+^ populations, would be pertinent effector cells in terms of rapid access to the final destination of the portal tract by extravasating through the vessel wall.

## Electronic supplementary material

Below is the link to the electronic supplementary material.

Supplementary material 1 (TIFF 2931 kb)

Supplementary material 2 (PDF 397 kb)
