# Supplementary figures and images for: Direct evidence for activated CD8+ T cell transmigration across portal vein endothelial cells in liver graft rejection
Source: J Gastroenterol. 2016 Feb 18;51(10):985–98. doi: 10.1007/s00535-016-1169-1 (PMC5037149; doi:10.1007/s00535-016-1169-1)

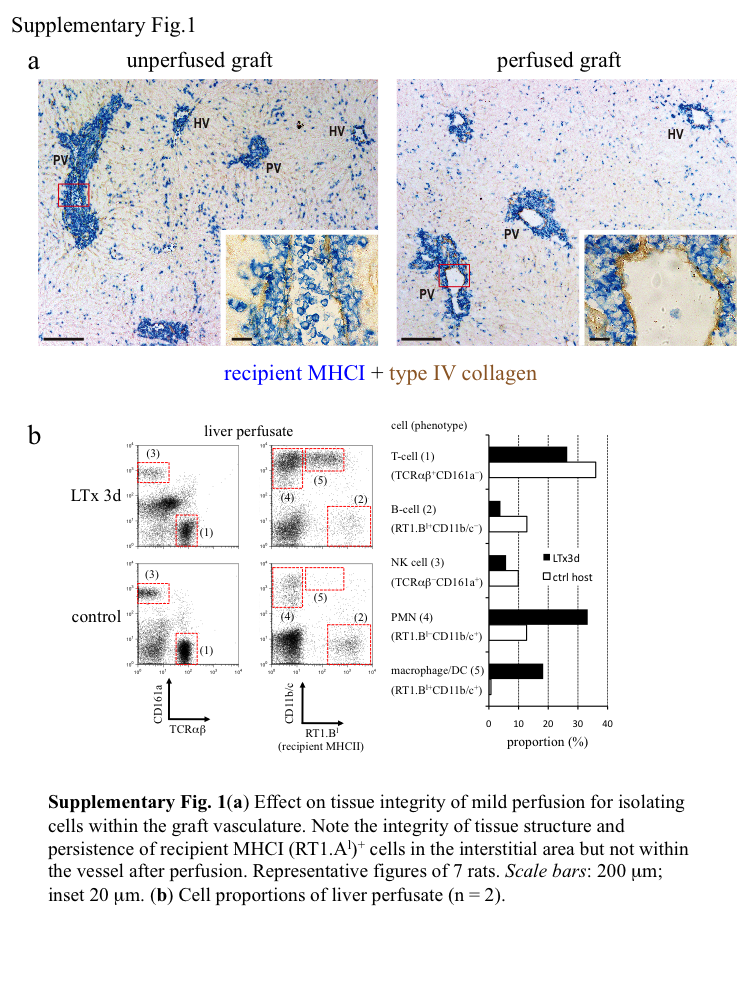

Supplement: Supplementary file 1 — Supplementary material 1 (TIFF 2931 kb) [file 535_2016_1169_MOESM1_ESM.tif]
