# Supplementary material for: Direct evidence for activated CD8+ T cell transmigration across portal vein endothelial cells in liver graft rejection
Source: J Gastroenterol. 2016 Feb 18;51(10):985–98. doi: 10.1007/s00535-016-1169-1 (PMC5037149; doi:10.1007/s00535-016-1169-1)
Supplement: Supplementary file 2 — Supplementary material 2 (PDF 397 kb) [file 535_2016_1169_MOESM2_ESM.pdf]

Supplementary Table1. Antibodies and probe used in this study

1st Ab

antigen	clone	subtype	source	conjugate (nil; purified)
CD8β	341	Ms IgG_1	AbD Serotec	fluorescein, Alexa flour 647[#]
CD11a (α_L integrin)	WT.1	Ms IgG_{2a}	Biolegend	R-phycoerythrin
CD11b/c (α_M integrin)	OX-42	Ms IgG_{2a}	Biolegend	R-phycoerythrin
CD15s (sialyl-6-sulfo Lewis X)	2H5	Ms IgM	provided by KR[1]	
CD18 (β2 integrin, LFA1β)	WT.3	Ms IgG_1	Cedarlane	fluorescein
CD25	OX-39	Ms IgG_1	Biolegend	R-phycoerythrin
CD29 (β1 integrin)	HMβ1-1	AHm IgG	Biolegend	R-phycoerythrin
CD31 (PECAM-1)	TDL-3A12	Ms IgG_1	AbD Serotec	Alexa flour 647
CD38	14.27	Ms IgG_{2b}	Biolegend	R-phycoerythrin
CD44H (H-CAM)	OX-49	Ms IgG_{2a}	Biolegend	fluorescein
CD49d (α4 integrin)	MRα4-1	Ms IgG_{2a}	BD Bioscience	fluorescein
CD54 (ICAM-1)	1A29	Ms IgG_1	Biolegend	R-phycoerythrin
CD62E (E-selectin)	polyclonal	Rb IgG	Biovision	
CD62L (L-selectin)	OX-85	Ms IgG_1	Biolegend	R-phycoerythrin
CD62P (P-selectin)	S789G	Ms IgM	provided by MS[2]	
CD106 (VCAM-1)	MR106	Ms IgG_1	Biolegend	
CD162 (PSGL-1)	polyclonal	Gt IgG	Santa Cruz	
CD163	ED2	Ms IgG_1	AbD Serotec	fluorescein[#]
CCR5	CTC5	Ms IgG_1	R&D	allophycocyanin
CCR9	CW-2.2.1	Ms IgG_{2b}	Santa Cruz	
CXCR3 (for flow cytometry)	868013	Ms IgG_{2b}	R&D	allophycocyanin
CXCR3 (for histochemistry)	XR3.2	AHm IgG	provided by TI[3]	
CXCR6	XR6.3	AHm IgG	provided by TI[3]	
CXCL10	polyclonal	Rb IgG	Peprotec	
tissue fibronectin	IST-9	Ms IgG_1	Santa Cruz	
β7 integrin	TA6	Ms IgG_1	provided by TI[3]	Alexa flour 647[#]
MAdCAM-1	OST2	Ms IgG_1	provided by MM[4]	
RT1.A^a (donor MHCI)	$MN_{4\text{-}91\text{-}6}$	Ms IgG_1	ECACC[*]	Alexa flour 488[#]
RT1.A^l (recipient MHCI)	I169.1	Ms IgG_{2c}	provided by HK[5]	
RT1.$B^{a/c}$ (donor MHCII)	OX-76	Ms IgG_{2a}	ECACC[*]	Alexa flour 488[#]
RT1.B^l (recipient MHCII)	OX-3	Ms IgG_1	ECACC	Alexa flour 647[#]
T-cell receptor αβ	R73	Ms IgG_1	ECACC[*]	PerCP Cy5.5[#]
VAP-1	174-5	Ms IgG_1	Abcam	
BrdU	BU1/75	Rat IgG_{2a}	AbD Serotec	
type IV collagen	polyclonal	Rb IgG	LSL	
type IV collagen-like structure	B12	Ms IgG_1	provided by TE[6]	

2nd Ab

product	source	conjugate
donkey IgG to goat IgG	R&D	allophycocyanin
donkey IgG to rabbit IgG	Jackson ImmunoResearch	alkaline phosphatase
donkey IgG to rat IgG	Jackson ImmunoResearch	alkaline phosphatase
goat IgG to A.hamster IgG	Jackson ImmunoResearch	alkaline phosphatase
goat IgG to mouse IgG	Sigma / Jackson ImmunoResearch	alkaline phosphatase / AMCA[$]
goat IgG to mouse IgG	BBI Solutions	10 nm colloidal gold
goat IgG to rabbit IgG	Invitrogen (Molecular Probes)	Alexa flour 594
goat $F(ab')_2$ to rabbit IgG	MP Bioscience	peroxidase
mouse IgG to fluorescein	Jackson ImmunoResearch	Alexa flour 488
rat IgG to mouse IgM	Biolegend	R-phycoerythrin

[1-6] generously provided by Drs. [1] R.Kannagi, [2] M.Suematsu, [3] TB.Issekutz, [4] M.Miyasaka, [5] H.Kimura, and [6] T.Ezaki, [$] 7-amino-4-methylcoumarin-3-acetic acid, [*] European Collection of Cell Cultures, [#] self conjugation

Abbreviations: Ms mouse, AHm Armenian Hamster, Rb rabbit, Gt goat
